# Using public clinical trial reports to probe non-experimental causal inference methods

**DOI:** 10.1186/s12874-023-02025-0

**Published:** 2023-09-09

**Authors:** Ethan Steinberg, Nikolaos Ignatiadis, Steve Yadlowsky, Yizhe Xu, Nigam Shah

**Affiliations:** 1 Center for Biomedical Informatics Research, Stanford University, Stanford, US; 2 Department of Statistics, University of Chicago, Chicago, US; 3 Google Research, Google, Cambridge, US

**Keywords:** Causal inference, Meta-analysis, Clinical trials, Method evaluation

## Abstract

**Background:**

Non-experimental studies (also known as observational studies) are valuable for estimating the effects of various medical interventions, but are notoriously difficult to evaluate because the methods used in non-experimental studies require untestable assumptions. This lack of intrinsic verifiability makes it difficult both to compare different non-experimental study methods and to trust the results of any particular non-experimental study.

**Methods:**

We introduce *TrialProbe*, a data resource and statistical framework for the evaluation of non-experimental methods. We first collect a dataset of pseudo “ground truths” about the relative effects of drugs by using empirical Bayesian techniques to analyze adverse events recorded in public clinical trial reports. We then develop a framework for evaluating non-experimental methods against that ground truth by measuring concordance between the non-experimental effect estimates and the estimates derived from clinical trials. As a demonstration of our approach, we also perform an example methods evaluation between propensity score matching, inverse propensity score weighting, and an unadjusted approach on a large national insurance claims dataset.

**Results:**

From the 33,701 clinical trial records in our version of the ClinicalTrials.gov dataset, we are able to extract 12,967 unique drug/drug adverse event comparisons to form a ground truth set. During our corresponding methods evaluation, we are able to use that reference set to demonstrate that both propensity score matching and inverse propensity score weighting can produce estimates that have high concordance with clinical trial results and substantially outperform an unadjusted baseline.

**Conclusions:**

We find that *TrialProbe* is an effective approach for probing non-experimental study methods, being able to generate large ground truth sets that are able to distinguish how well non-experimental methods perform in real world observational data.

## Background

Non-experimental studies (which are also known as observational studies) are valuable for estimating causal relationships in medical settings where randomized trials are not feasible due to either ethical or logistical concerns [1]. In addition, effects from randomized trials might not generalize to real-world use due to limited and non-representative study populations and differing clinical practice environments [2]. Accurately estimating these causal relationships is important, as learning which treatments are the most effective is a key component of improving health care. However, non-experimental studies are difficult to use in practice due to the absence of randomization, which forces them to rely on difficult-to-verify assumptions, such as the absence of unmeasured confounding and non-informative censoring [3]. These assumptions make it difficult to evaluate the performance of non-experimental methods, which is an important step for verifying the reliability of these techniques as well as determining the relative merits of different methods. Despite significant recent progress in non-experimental study evaluation (detailed in Section “Related work”), this difficulty with evaluation hampers research, by making it more difficult to develop more effective methods, and hinders practice, as clinicians are hesitant to use evidence generated from non-experimental studies even in situations where clinical trial derived evidence is not available [4–6].

In this work, we introduce *TrialProbe*, a new principled approach for the systematic appraisal of non-experimental causal inference methods. Our basic premise is that we can evaluate non-experimental causal inference methods by comparing adverse event effect estimates from non-experimental methods with published experimentally derived estimates from public ClinicalTrials.gov clinical trial reports. Compared to previous approaches for the evaluation of non-experimental methods (more of which below in Section “Related work”), *TrialProbe* differs in three regards. First, we explicitly focus on active comparator study designs where one drug is directly compared to another drug as those are easier to connect to potential non-experimental study designs [7]. Second, we estimate the magnitude of the effects extracted from the public clinical trial reports through an empirical Bayes approach that explicitly accounts for the heterogeneity of odds ratios across the clinical trials, the statistical information content (e.g., sample size) used to estimate each odds ratio, and the fact that most effects are very small. Third, we use those estimated effects to split our reference set into several subsets that contain drug effects of varying strengths, so that users can simultaneously understand the concordance between non-experimental and experimental methods for both stronger and weaker effects.

We then use *TrialProbe* to evaluate common non-experimental study methods in terms of their ability to identify causal relationships from a large national administrative claims dataset - Optum’s de-identified Clinformatics Data Mart Database. We find that available methods can reproduce a significant fraction of the reported effect and that adjusting for a low-dimensional representation of patient history outperforms a naive analysis that does not adjust for any covariates.

### Related work

The importance of evaluating non-experimental methods is well-understood and ubiquitous. The most common approach for evaluation is based on simulation experiments, or more recently, based on semi-synthetic simulations that seek to mimic real observational datasets [8–12]. The upshot of simulation studies is that the ground truth is precisely known, and so non-experimental methods can be compared with respect to any metric of interest. Nevertheless, it is difficult to determine whether or not those simulations provide a realistic confounding structure that is similar to observational data in practice.

Non-experimental methods have also been evaluated in terms of reproducibility by evaluating whether it is possible to independently reproduce previously published non-experimental studies [13]. Reproducibility is an important and useful feature for non-experimental studies, but measuring reproducibility alone does not necessarily address the issue of whether non-experimental studies provide correct effect estimates.

Closer to our work, several authors have evaluated non-experimental methods by comparing them to results from RCTs. Some authors have used data from RCTs to estimate a causal effect, and then applied a non-experimental method only to the treatment arm of the same RCT [14, 15]^1^ or to the treated subjects from the RCT along with control subjects drawn from survey datasets [16]. Furthermore, such approaches require access to patient-level data for each RCT.

Other authors have constructed pairs of published non-experimental studies and RCTs that assess the same intervention in similar populations [17, 18]. Such an approach is appealing, as it directly compares non-experimental designs that researchers have pursued (and published). On the other hand, such an approach does not allow the large-scale and systematic exploration of variations in causal inference methods and is typically restricted to the study of dozens of effects. This approach is also subject to publication bias issues, which results in an under-reporting of non-significant effects in both experimental and non-experimental designs.

Another common approach—that most closely aligns with our work—for evaluating non-experimental causal inference methods is through reference sets [19, 20]. A reference set is a collection of relationships about the effects of treatments that are independently verified, and treated as ground truth against which the ability of a non-experimental method to identify those effects from available data can be quantified. There have been several proposed approaches to create reference sets, the most prominent of which rely on either FDA labels or expert knowledge to declare known relationships between drugs and outcomes [20]. However, the actual construction of existing reference sets can be opaque. Instead, in *TrialProbe* we generate a sequence of nested reference sets that correspond to increasing levels of evidence for the strength of the causal effect. The construction of the *TrialProbe* reference sets is fully data-driven and reproducible. Furthermore, we are not aware of previous reference sets that focus on active comparator study designs.

RCT-Duplicate [21] is another closely related effort that attempts to quantify the performance of non-experimental methods by carefully reproducing the results of 32 clinical trials using insurance claims databases. This manual emulation of the trial design (to the extent feasible) allows RCT-Duplicate to very closely match the exact clinical trial setup, including details such as inclusion/exclusion criteria that are not possible with fully automated approaches such as ours. In addition, the increased effort per trial limits the number of RCTs that can be feasibly reproduced to just 32. Our work is similar in spirit, but expands on the idea by vastly increasing the number of estimated effects by several orders of magnitude to 12,967 by being fully automated and by taking advantage of the entire ClinicalTrials.gov database.

All the approaches we outlined above for the evaluation of non-experimental methods based on results from RCTs face the following difficulty: Even in an optimal situation, it is not expected that any non-experimental method will reproduce the entire ground truth in the reference set because the observational data usually comes from a different population than the population used to collect the ground truth [22]. Identification of a known relationship might fail for example because the academic medical center population used in an RCT might differ drastically from the general population available in the non-experimental data resource. Many other study design factors (e.g., whether the estimand is a hazard ratio in the non-experimental study and an odds ratio in the RCT) can further lead to deviations between the non-experimental study and the RCT. A related issue is that experimental studies also have a certain error rate, in that incorrect blinding, randomization, unrealistic usage, or other errors can cause an RCT to return incorrect effect estimates [2]. Nevertheless, a common assumption is that while the exact effect might differ, the effect identified in the observational data and the original “ground truth” should be correlated and good non-experimental methods should on average have greater correspondence with the provided ground truth [23]. Here we take this idea to an extreme and only check for concordance between the direction of effects in RCTs and the non-experimental methods [12, 20]. A related evaluation approach, where one only seeks to recover the direction of an effect, has appeared in the causal discovery literature [24].

## Methods

In this section we describe the *TrialProbe* approach. We describe the data source of the clinical trial reports (ClinicalTrials.gov), the processing of the raw data to a curated dataset of $M={12,967}$ unique drug/drug adverse event comparisons, as well as the statistical approach that we propose for comparing non-experimental causal inference methods.

### The primary data source: ClinicalTrials.gov

ClinicalTrials.gov serves as a public repository for clinical trials carried out in the United States and abroad. The database contains pre-registration information, trial status, and results as provided by researchers conducting the trials. Many clinical trials are legally required to report results to ClinicalTrials.gov within 1 year of study completion, with a compliance rate of over 40%  [25]. In this work we use the June 4, 2020 version of the database, which includes 33,701 clinical trials. Note that we are not using patient level data collected in the trial, but the public report posted at ClinicalTrials.gov.

### Extracting trials with an active comparator design

We focus on drug versus drug active comparator clinical trials, which evaluate one drug directly against another. The reason is that such comparisons are easier to conduct in the context of a non-experimental study design. In contrast, placebo or standard of care based trials are more difficult to work with because there is no clear corresponding case-control non-experimental study that can be used to estimate effects. We additionally restrict our analysis to higher quality clinical trials using the study design reported on ClinicalTrials.gov. We implement a quality filter by inspecting the reported randomization and blinding information and explicitly removing trials that are either not randomized or do not use participant blinding.

The results section of each active comparator clinical trial record consists of a set of intervention arms as well as the primary outcomes and adverse events associated with each arm. The primary outcomes and side effects are all specified in natural language and must be mapped to standardized terminologies. We discard the primary outcomes because it is difficult to consistently map them to electronic healthcare data sources due to a wide diversity of measurements and a lack of standardized terminology. We instead focus on the adverse events because they are specified using MedDRA terminology and because mappings to corresponding condition codes are available for healthcare data sources. We obtain a standardized version of these adverse outcomes by mapping them to ICD10 using the dictionary mappings contained within UMLS 2019AB.

The drug mentions in the ClinicalTrials.gov records are specified in an ad-hoc manner in terms of brand names, ingredients, dosages and/or more specialized names. As a preliminary step, we filter out all treatment arms with fewer than 100 patients as trials of that size frequently do not have enough power to obtain statistical significance. We then use the RxNorm API to transform the text descriptions of drugs into RxNorm ingredient sets. We require at least 50% of the tokens to match in order to avoid false positives. Treatment arms with more than one ingredient (due to either containing multiple drugs or drugs with multiple active ingredients) are also filtered out. As an additional quality control step, we remove intervention arms that contain plus (“$+$”) signs in their names that usually indicate combination treatments that RxNorm is not always able to detect and map to ingredients correctly. Finally, we map those RxNorm ingredient sets to Anatomical Therapeutic Chemical (ATC) codes so that we can find the corresponding drugs more easily in our ATC code annotated observational data. We manually very that this automated drug name extraction and mapping step did not introduce significant errors by manually inspecting a set of 100 random mapped trials and double-checking that all drugs in those trials were resolved to correct the RxNorms.

One important feature of ClinicalTrials.gov data is that it often contains records where the same drug-drug comparisons have been tested in multiple trials. We aggregate side effect event counts and participant counts for trials with identical drug combinations and outcome measurements. Similarly, we also aggregate counts across arms where the same drug was evaluated with different dosages. This aggregation procedure has the dual purpose of strengthening the reliability of consistent true effects while helping to down-weigh trials with conflicting effects.

We also note that in an active comparator design, there is typically no concrete choice for the baseline arm (in contrast to e.g., placebo or standard of care trials)—the role of the two arms is *symmetric*. To express this symmetry, we reorder all pairs of drugs under comparison (for each adverse event) in such a way that the sample odds ratio is $\ge 1$.

At the end of this process, we have compiled $M={12,967}$ unique drug versus drug treatment adverse event comparisons. The summarized data for the *i*-th entry comprises of the ICD10 code of the adverse event, the ATC code of the two drugs being compared, as well as the contingency table $Z_i$:

1 $$\begin{aligned} Z_i = \begin{array}{lcc} &{} \text {Drug A} &{} \text {Drug B} \\ \text {Number of patients with the adverse event} &{} X_{A,i} &{} X_{B,i} \\ \text {Number of patients without the adverse event} &{} Y_{A,i} &{} Y_{B,i} \\ \end{array} \end{aligned}$$

Below we describe our concrete statistical proposal for leveraging the above dataset to compare non-experimental causal inference methods.

### Empirical Bayes effect size estimation

In this section, we develop an approach for estimating the effect sizes of all the drug versus drug treatment adverse event comparisons that adjusts for the following issues: First, most of the drug vs drug effect sizes are very small, close to 1, if not non-existent. Adjusting for this prior is necessary in order to reject spurious, but statistically significant effects. Second, each drug vs drug comparison contains vastly different amounts of information, with differing event rates, patient counts, etc for each comparison. Taking into account the differences in information content is important for identifying effects that are weak, but strongly supported due to the quantity of clinical trial evidence.

Our estimation approach follows a tradition of methodological developments based on hierarchical modeling combined with an empirical Bayes analysis [26–29]. This approach explicitly learns a prior to take into account how most effects are small and takes advantage of the differing amounts of information in each comparison. We model the likelihood for the log odds ratio $\omega _i$ of the *i*-th comparison (with contingency table (1)) through the non-central hypergeometric distribution, that is,

2 $$\begin{aligned} L_i(\omega _i)=\frac{\left( {\begin{array}{c}X_{A,i} + Y_{A,i}\\ X_{A,i}\end{array}}\right) \left( {\begin{array}{c}X_{B,i} + Y_{B,i}\\ X_{B,i}\end{array}}\right) \textrm{e}^{\omega _i X_{A,i}}}{\sum _{t}\left( {\begin{array}{c}X_{A,i} + Y_{A,i}\\ t\end{array}}\right) \left( {\begin{array}{c}X_{B,i} + Y_{B,i}\\ X_{A,i} + X_{B,i} - t\end{array}}\right) \textrm{e}^{\omega _i t}} \end{aligned}$$

The likelihood $L_i(\omega _i)$ for the analysis of $2 \times 2$ contingency tables has been proposed by, e.g., [30–33], and is derived by conditioning on the margins of the table $Z_i$—in entirely the same way as in the derivation of Fisher’s exact test.

In our hierarchical approach, we further model the $\omega _i$ as exchangeable random effects, independent of the margins of $Z_i$, with:

3 $$\begin{aligned} \omega _i\, (i=1,\dotsc ,M) \, \,{\buildrel \text {iid} \over \sim \,}\, G, \;\;\; \text {G is a symmetric distribution around the origin} \end{aligned}$$

In contrast to a fully Bayesian approach, we do not posit knowledge of *G*, but instead follow the empirical Bayes paradigm and estimate *G* based on the data $Z_1,\dotsc ,Z_M$ as follows:

4 $$\begin{aligned} \widehat{G} \in \underset{G}{\textrm{argmax}} \left\{ \sum _{i=1}^M \log \left( { \int L_i(\omega ) dG(\omega )}\right) \;:\; \text {G is a symmetric distribution around }0\right\} \end{aligned}$$

Equation (4) is an optimization problem over all symmetric distributions *G* and the objective is the marginal log-likelihood—each component likelihood $L_i(\cdot )$ (2) is integrated with respect to the unknown *G*. The estimator $\widehat{G}$ is the nonparametric maximum likelihood estimator (NPMLE) of Kiefer and Wolfowitz  [34], and has been used for contingency tables [30]. We note that in contrast to previous works  [30], we also enforce symmetry of *G* around 0 in (3), (4). The reason is that, as explained in Section “Extracting trials with an active comparator design”, our active comparator design setting is symmetric with respect to the drugs under comparison.

**Fig. 1:**
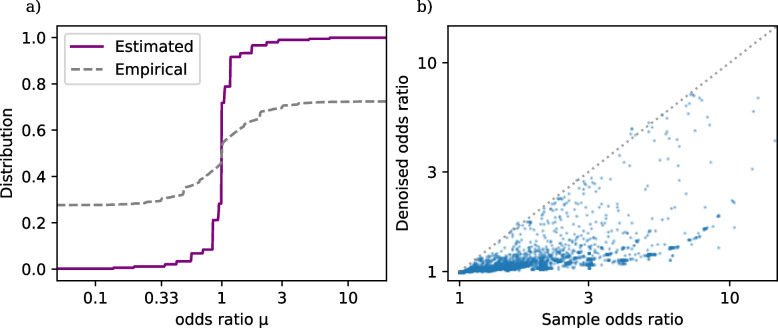
**a** Distribution function of drug versus drug adverse event odds ratios in *TrialProbe*. $\widehat{G}$ is estimated via nonparametric maximum likelihood as in (4), while the dashed curve is the empirical distribution of sample odds ratios. **b** Denoised vs. raw odds ratios. Denoising (5) is done by computing the posterior mean of the log odds ratio given the data for the *i*-th comparison and the estimated $\widehat{G}$

Figure 1a shows the estimated distribution function $\widehat{G}$ (4) based on the *TrialProbe* dataset (in terms of odds ratios $\textrm{exp}(\omega _i)$, but with a logarithmic *x*-axis scale), as well as the empirical distribution of sample odds ratios.^2^ We observe that even though the sample odds ratios are quite spread out, the NPMLE $\widehat{G}$ is substantially more concentrated around odds ratios near 0. This is consistent with the intuition that for an active comparator design study, side effects will often be similar for the two drugs under comparison (but not always).

Finally, to create an effect estimate for the the drug versus drug treatment adverse event comparisons, we use the plug-in principle: We use the estimated $\widehat{G}$ to compute denoised point estimates of the log odds ratios via the empirical Bayes rule :

5 $$\begin{aligned} \widehat{\omega }_i^{\text {EB}}= \mathbb {E}_{\widehat{G}}[{\omega _i \mid Z_i}] = \frac{\int \omega L_i(\omega ) d\widehat{G}(\omega )}{\int L_i(\omega ) d\widehat{G}(\omega )} \end{aligned}$$

Figure 1b plots $\textrm{exp}(\widehat{\omega }_i^{\text {EB}})$ against the sample odds ratios. We observe that the rule $\widehat{\omega }_i^{\text {EB}}$ automatically shrinks most sample log odds ratios toward 0 (equivalently: $\textrm{exp}(\widehat{\omega }_i^{\text {EB}})$ shrinks most sample odds ratios toward 1), while rigorously accounting for varying effective sample size of each comparison (so that shrinkage toward 1 is heterogeneous). Table 1 gives the first ten entries of *TrialProbe*, with the largest denoised odds ratio $\textrm{exp}(\widehat{\omega }_i^{\text {EB}})$.

### Effect size ranking and subsetting

Given our effect size estimates computed through empirical Bayes, we rank drug vs drug adverse event comparisons by effect size magnitude [35] and construct subsets of our reference set that only contain effects greater than a chosen magnitude.

There is a challenging trade-off when choosing the effect size threshold required to be included in the reference set. Stronger effects should be more resilient to errors in either the clinical trial or non-experimental study design, but might exclude moderate effects that clinicians and researchers are interested in estimating with non-experimental methods.

Due to that complicated trade-off, we do not choose a static effect size threshold and instead perform all analyses with all possible effect size thresholds. This strategy also allows us to provide some insight into how metrics degrade as weaker effects are allowed in the reference set.

We thus define a family of reference sets $S_t$, where *t* is the minimum required denoised odds ratio to be included in the set. Each set $S_t$ is a subset of *TrialProbe*, defined as follows:

6 $$\begin{aligned} \mathcal {S}_t = \left\{ i \in \textit{TrialProbe}\,:\, \textrm{exp}(\widehat{\omega }_i^{\text {EB}}) \ge t\right\} \end{aligned}$$

**Table 1:** Example *TrialProbe* entries. The 10 entries of our reference set with the largest denoised odds ratio. Each entry corresponds to a drug vs. drug (columns 2 and 3) comparison with respect to an adverse event (column 1). We also report the NCT of the clinical trial(s) that furnish the experimental evidence for the comparison (column 4), the summarized data from the RCT in the form of a contingency table as in (1) (column 5) and the denoised odds ratio $\textrm{exp}(\widehat{\omega }_i^{\text {EB}})$ (5) (column 6)

Adverse Event (ICD10)	Drug A (ATC)	Drug B (ATC)	Clinical Trial NCTs	Contingency Table	Denoised Odds Ratio
Hiccups (R06.6)	Nicotine (N07BA01)	Bupropion (N06AX12)	332644	$$\left[ \begin{array}{rr} 0 &{} 264 \\ 35 &{} 225 \\ \end{array}\right]$$	58.63
Dry Mouth (K11.7)	Bupropion (N06AX12)	Nicotine (N07BA01)	332644	$$\left[ \begin{array}{rr} 0 &{} 260 \\ 20 &{} 244 \\ \end{array}\right]$$	27.30
Conjunctivitis (H10)	Brimonidine (D11AX21)	Brinzolamide (S01EC04)	1297920	$$\left[ \begin{array}{rr} 0 &{} 234 \\ 15 &{} 220 \\ \end{array}\right]$$	15.56
Nausea (R11.0)	Dulaglutide (A10BJ05)	Glimepiride (A10BB12)	1644500	$$\left[ \begin{array}{rr} 1 &{} 242 \\ 25 &{} 219 \\ \end{array}\right]$$	12.82
Dysgeusia (R43.2)	Brinzolamide (S01EC04)	Brimonidine (D11AX21)	1297920 1297517	$$\left[ \begin{array}{rr} 2 &{} 453 \\ 38 &{} 422 \\ \end{array}\right]$$	11.02
Heartburn (R12)	Nicotine (N07BA01)	Bupropion (N06AX12)	332644	$$\left[ \begin{array}{rr} 2 &{} 262 \\ 31 &{} 229 \\ \end{array}\right]$$	8.52
Vomiting (R11.1)	Dulaglutide (A10BJ05)	Glimepiride (A10BB12)	1644500	$$\left[ \begin{array}{rr} 1 &{} 242 \\ 19 &{} 225 \\ \end{array}\right]$$	7.60
Hypoglycaemia (E16.2)	Glipizide (A10BB07)	Sitagliptin (A10BH01)	86515 94770 509262	$$\left[ \begin{array}{rr} 61 &{} 1201 \\ 279 &{} 754 \\ \end{array}\right]$$	7.10
Dysgeusia (R43.2)	Telavancin (J01XA03)	Vancomycin (A07AA09)	107978 91819	$$\left[ \begin{array}{rr} 62 &{} 876 \\ 311 &{} 618 \\ \end{array}\right]$$	7.06
Nausea (R11.0)	Tigecycline (J01AA12)	Ertapenem (J01DH03)	366249	$$\left[ \begin{array}{rr}46 &{} 462 \\ 233 &{} 320 \\ \end{array}\right]$$	6.97

### Evaluation: concordant sign rate

As explained previously, there are many possible reasons why the exact effect size from a non-experimental assessment of a causal effect may not match the results of a clinical trial. We propose to handle this by only looking at the estimated effect direction for those effects which are known to be large. We additionally only compare concordance for cases where the non-experimental method returns a statistically significant result, as this both removes cases where we wouldn’t expect the non-experimental assessment to match and better aligns with how non-experimental assessments are used in practice. The basic premise of our approach is the following.

Consider the comparison of two drugs with respect to an adverse event. Suppose that:

1. In the clinical trial report, there is *strong* evidence that $\omega _A \gg \omega _B$, that is, there is strong evidence that the adverse event rate under drug A is * substantially larger* compared to drug B.
2. The non-experimental causal inference method yields a significant *p*-value, indicating that the null hypothesis (that both drugs have the same adverse event rate) is probably false.
3. According to the non-experimental method, drug B leads to a higher adverse event rate compared to drug A, that is, the direction of the effect is the opposite compared to the clinical trial evidence.

Then, we are confident that the non-experimental method yields misleading evidence in this case as it provides statistically significant effects in the wrong direction compared to the ground truth.

We instantiate the above framework as follows. We seek to systematically evaluate a non-experimental causal inference method $\mathcal {O}$, which we define as follows (see Section “Case study on Optum’s Clinformatics” for a concrete instantiation): $\mathcal {O}$ is a mapping from two drugs (drug A and drug B) and an adverse event to a *p*-value and a predicted causal effect direction (i.e., whether drug A or drug B causes the adverse event more frequently). Specifying the mapping $\mathcal {O}$ requires specification of the healthcare data resource, the protocol for extracting subjects treated with drug A, resp. drug B, and a statistical method (e.g., an observational method that adjusts for observed covariates) that returns a *p*-value and the predicted effect direction.

We define $\mathcal {R}(\mathcal {O}) \subset \textit{TrialProbe}$ as the set of comparisons such that the non-experimental study returns a *p*-value $\le 0.05$. In order to ensure that we only evaluate larger effects, we use the $S_t$ subsets of *TrialProbe* defined in the previous section which require each entry in the set to have an empirical Bayes denoised odds ratio greater than *t*.

We then define the Concordant Sign Rate, as:

7 $$\begin{aligned} \text {CSR}(\mathcal {S}_t, \mathcal {O}) = \frac{ \#\left\{ i\in \mathcal {S}_t\cap \mathcal {R}(\mathcal {O}): \text {Direction of effect for}\ i\ \text{agrees between } \mathcal {S}_t,\mathcal {O}\right\} }{ \# \{i\in \mathcal {S}_t\cap \mathcal {R}(\mathcal {O})\}} \end{aligned}$$

Large values of $\text {CSR}(\mathcal {S}_t, \mathcal {O})$ are preferable. We may define $1-\text {CSR}(\mathcal {S}_t, \mathcal {O})$ as the discordant sign rate, which is analogous to the notion of false sign rate in multiple testing [36, 37] and the type-S (“sign”) error [38]. In the present setting, however, there is no precise notion of “true” and “false” sign, and instead we evaluate only based on concordance/discordance with the effect derived from the public clinical trial reports.

For every $\mathcal {S}_t$ and every non-experimental causal inference method $\mathcal {O}$, we compute two metrics: the fraction of statistically significant results that have a concordant sign (as in (7)) and the fraction of entries of $\mathcal {S}_t$ recovered (as in being marked statistically significant with concordant sign). The concordant sign rate gives an indication of how reliable a non-experimental method is and the fraction recovered gives an indication of its power.

### Case study on Optum’s Clinformatics

To illustrate how *TrialProbe* may be applied, we consider a hypothetical investigator who is interested in comparing two drugs with respect to a specific adverse event and seeks to generate evidence for the comparison. The investigator has access to Optum’s de-identified Clinformatics Data Mart 8.0 medical claims dataset [39], a large US commercial claims dataset containing over 88 million patients that is frequently used for non-experimental studies.

The investigator proceeds as follows:

1. Cohorts are constructed systematically using the first drug reimbursement claim for either of the two drugs as the index time. Patients with a prior event or an event at the index time are excluded. At most 100,000 patients are sampled for each drug. Outcomes are measured until each record is censored (as indicated by the end of their healthcare enrollment in the Clinformatics dataset).
2. For the cohort generated as above, the investigator fits a Cox proportional hazards model with response equal to the first time the adverse event occurs and covariate equal to the indicator of treatment assignment to drug A.^3^
3. The investigator reports a significant causal effect if the *p*-value from the Cox fit is $\le 0.05$ and in that case, declares the direction of the effect according to the estimated hazard ratio.

Steps 1—3 comprise a non-experimental strategy $\mathcal {O}$. We also consider two additional non-experimental strategies that replace step 2. by 2.’ or 2.”:

2.’ The investigator fits a propensity score matched (PSM) Cox model. The propensity score is estimated using logistic regression on a low-dimensional representation of the patient’s history obtained via a procedure by Steinberg et al. [40]. When performing matching, the investigator uses a 1:1 greedy matching algorithm on the logit scale with a caliper of 0.1. Once a matched cohort is chosen, the hazard ratio is estimated using a Cox regression by modeling the survival outcome as a function of the treatment status in the cohort. The calculation of the *p*-value corresponding to the hazard ratio ignores the estimation of the propensity scores.
2.” The investigator fits an inverse propensity score weighted (IPSW) Cox model. As in 2.’, the propensity score is estimated using logistic regression on a low-dimensional representation of the patient’s history obtained via a procedure by Steinberg et al. [40]. The calculation of the *p*-value corresponding to the hazard ratio ignores the estimation of the propensity scores.

In what follows, we refer to these two non-experimental methods as “Unadjusted Cox”, “Cox PSM” and “Cox IPSW”. We note that there are many possible criticisms to all three approaches. For example, the first approach is naïve, in that it does not even attempt to adjust for confounding. The second approach adjusts for confounding, but also has caveats, e.g., the computed standard error may be overly conservative [41]. Finally, the third approach, IPSW, has relatively high variance and can be unstable, especially when there is minimal overlap. Nevertheless, it is plausible that an investigator would proceed using one of these non-experimental approaches (especially Cox PSM and Cox IPSW). With *TrialProbe*, we can probe some of the properties of these three non-experimental methods.

For a given comparison of interest, it could be the case that any of the methods provides more reliable evidence than the others, or perhaps all methods provide unreliable evidence. There are many reasons why the methods could fail to provide reliable evidence, and these reasons may vary from comparison to comparison (as explained before). Through *TrialProbe* we probe operating characteristics of methods in *aggregate* over many possible comparisons. At the same time, we also encourage researchers to delve in more depth at specific comparisons to identify failure modes of non-experimental strategies.

## Results

From the 12,967 unique drug vs drug treatment adverse event comparisons that we were able to extract from ClinicalTrials.gov, 1,124 of them have fewer than 100 patients for each drug in Optum’s Clinformatics and are discarded due to lack of data. That leaves 11,843 comparisons with sufficient patients in Optum’s Clinformatics to estimate treatment effects. To illustrate how *TrialProbe* proceeds, we first consider the 10 strongest effects according to the empirical Bayes ranking described in Section “Empirical Bayes effect size estimation”. Each row of Table 2 corresponds to one drug vs. drug adverse event comparison, and the first columns correspond to the AE, the two drugs under comparison. The last three columns correspond to the non-experimental evaluation. For all methods we show the *p*-value, and the direction of the effect (i.e., whether Drug A or Drug B leads to more severe AE). When the *p*-value is significant ($\le 0.05$), then we also color the cell as  , resp.  depending on whether the directionality of the effect is  , resp.  .

**Table 2:**
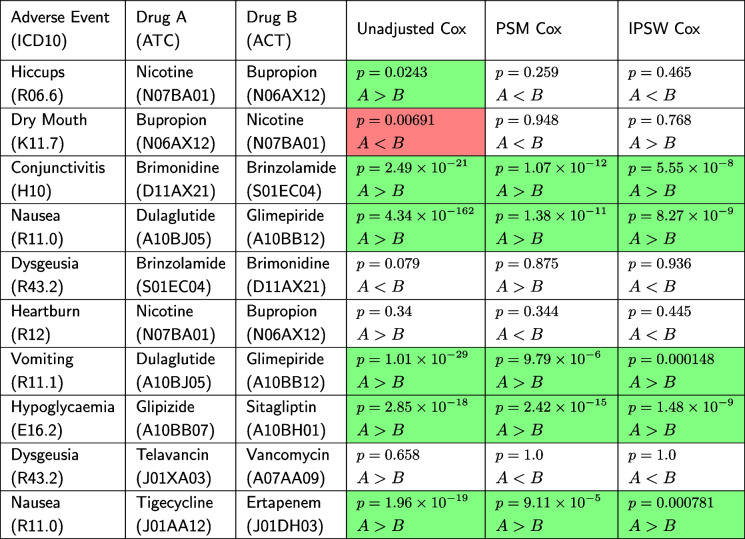
Example *TrialProbe* analysis. The rows correspond to the same 10 comparisons as the rows of Table 1 and the first 3 columns are identical. The last 3 columns correspond to each of the three non-experimental strategies considered and show the *p*-value returned from each method for each comparison, as well as the direction of the effect. Cells demarcated by green correspond to significant effects with detected directions that are concordant with the RCT result, while red denotes a discordant significant effect

As an example, the effect in the third row is so strong, so that all three non-experimental methods declare the effect as significant and determine a concordant direction. On the other hand, we do not see good concordance or recovery for the Nicotine vs Bupropion examples (rows one, two, and six), with the covariate-adjusted methods returning three statistically insignificant results and the unadjusted method returning one statistically significant concordant result, one statistically significant discordant result, and one statistically insignificant result. This illustrates some of the tradeoffs when adjusting for confounders in that adjusted methods have an increased Type 1 error rate, but also an increased Type 2 error rate. A likely explanation for the poor performance with nicotine in particular is that nicotine usage is frequently not recorded well in claims data. In this case the potential mismatch between trial results and non-experimental results may be more due to the data source, and not due to the adjustment strategies. This example thus illustrates how *TrialProbe* can help identify failure modes of non-experimental studies.

**Fig. 2:**
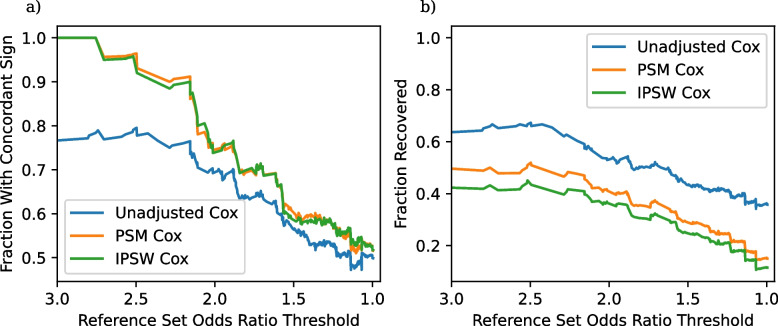
**a** Fraction of significant results with concordant sign as a function of the odds ratio threshold in (6). **b** Fraction of recovered entries as a function of the odds ratio threshold

We continue with a more holistic picture of the comparison of the two non-experimental strategies (instead of looking at results for individual comparisons) and proceed as suggested in Section “Evaluation: Concordant sign rate”. One important aspect of our results is that many of the non-experimental effect estimates are not statistically significant, and thus not evaluated by our pipeline. The fraction of non-significant results are in Table 3. The high frequency of non-significant results, even with the use of a large observational dataset probably reflects the fact that many of these adverse events are rare, especially given the underreporting common in claims data. We compute the fraction of significant results that have concordant signs and the fraction of reference set entries correctly recovered by each method for each subset $S_t$ of *TrialProbe* that only contains effects that have an odds ratio threshold greater than *t*. Figure 2 provides the performance of each of our three methods on these two metrics. It is reassuring that for the relatively strong effects, all methods perform better than a “coin-flip” based guess of directionality. On the other hand, also as anticipated, the methods that adjust for confounders have better concordance compared to unadjusted Cox-PH and the concordant sign rate is $\ge 80\%$ for comparisons with strong evidence in ClinicalTrials.gov, say, with (denoised) odds ratio $\ge 2$.

**Table 3:** Percentage of non-experimental results where were statistically significant at $p < 0.05$ for each evaluated non-experimental method

	Unadjusted Cox	PSM Cox	IPSW Cox
% Stat. Significant	71.67%	29.05%	22.19%

We make the following remarks: As the *x*-axis varies in the plots, we are scanning over less stringent choices of “reference sets”. However, in the spirit of probing methods in an exploratory way, we do not need to make a choice of a specific reference set / cutoff on the *x*-axis. We also note that as the denoised odds ratios approaches zero, the “reference set” $\mathcal {S}_t$ becomes increasingly uninformative, and so we would anticipate that *any* method would have $\text {CSR} \approx 0.5$.

### Comparison to prior work

In order to better understand how *TrialProbe* compares to prior work, we perform three other non-experimental method evaluation strategies. First, we perform a direct concordance and recovery rate evaluation using the positive controls (that are presumed to have an effect) from the OMOP and EU-ADR reference sets. We also create an ablated form of *TrialProbe* that does not use the empirical Bayesian effect estimation and odds ratio magnitude filtering, and instead only identifies significant effects using an exact Fisher test with a 0.05 *p*-value threshold. Table 4 contains the results of this comparison.

**Table 4:** Performance of non-experimental methods according to the EU-ADR and OMOP reference sets with a comparison baseline a *TrialProbe* subset that consists of all entries that are statistically significant according to the Fisher exact test instead of an empirical Bayes odds ratio threshold

Non-experimental	OMOP (*n*=140)		EU-ADR (*n*=32)		Fisher exact test *TrialProbe* Subset (*n* = 293)	
Method	% Concordant	% Recovered	% Concordant	% Recovered	% Concordant	% Recovered
Unadjusted Cox	50 %	45 %	35 %	22 %	53 %	42 %
PSM Cox	47 %	20 %	60 %	9 %	59 %	30 %
IPSW Cox	65 %	65 %	53 %	53 %	58 %	27 %

We find that all three of these sets, OMOP, EU-ADR, and the corresponding *TrialProbe* subset that only required Fisher statistical significance, were difficult to reproduce, with many non-concordant signs and lost effects. The low concordance and recovery of Fisher exact test based *TrialProbe* subset in particular helps indicate the importance of our empirical Bayesian estimation and effect size filtering.

### Importance of clinical trial filtering

**Fig. 3:**
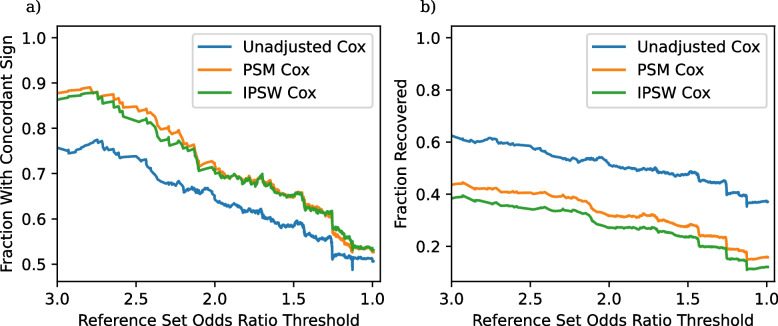
Concordance and recovery rates for an ablated form of *TrialProbe* that does not use clinical trial quality filters. **a** Fraction of significant results with concordant sign as a function of the odds ratio threshold in (6). **b** Fraction of recovered entries as a function of the odds ratio threshold

One of the key decisions for constructing *TrialProbe* is which clinical trials to include for analysis. Our analysis uses an assignment and blinding filter, requiring all candidate clinical trials to use randomized assignment and participant blinding. This filter excludes 6,855 of the 19,822 candidate effects that we could have otherwise studied. In order to understand the effect of this filter, and whether it is worth the lost entries, we perform an ablation experiment where we rerun our analysis without this filter. The resulting concordance and recovery plots are in Fig. 3.

The concordance rate and recovery rate without the clinical trial quality filter are distinctly lower, especially at larger odds ratio thresholds. This probably reflects how low-quality clinical trials are less likely to be reproducible due to the inherent increased error rate caused by a lack of participant blinding and incomplete randomization.

## Discussion

In this work, we use clinical trial records from ClinicalTrials.gov to build a source of ground truth to probe the performance non-experimental study methods. We show how such a dataset can be constructed in a systematic statistically sound manner in a way that also allows us to filter by the estimated strength of the effects. We also demonstrate the value of our approach by quantifying the performance of three commonly used non-experimental study methods.

Our approach has three advantages. First, it characterizes the performance of methods on real observational data. Second, our approach provides high quality ground truth based on clinical trials that have varying effect sizes, allowing a read out of the performance of a method for a given effect size (Fig. 2). Prior reference sets rely on ground truth sources that might be less reliable or have weaker relationships. Finally, our approach scales better than prior work, because we can create thousands of “known relationships” from published trial reports. This is a significant advantage compared to prior approaches that rely on evaluating methods using patient-level randomized trial datasets that can be difficult to acquire [42].

The empirical Bayes estimation and odds ratio magnitude subsetting in particular seems to be a key component of how *TrialProbe* can achieve relatively high measured concordance between the clinical trials and non-experimental methods. As shown in our results section, a *TrialProbe* subset that only relies on statistical significance achieves very low concordance. Likewise, the OMOP and EU-ADR reference sets (which indirectly rely only on statistical significance through FDA reports) also report similarly poor performance. We believe the most likely hypothesis for explaining this is that there is likely to be significant type 1 error due to the implicit vast multiple hypothesis testing problem when searching for a small number of significant adverse event effects in a sea of thousands of reported minor effects. Empirical Bayes automatically adjusts for this multiple hypothesis testing issue by learning a prior that incorporates the knowledge that most adverse event effects are null (Fig. 1), and can thus more effectively discard these invalid effects.

However, our approach has several limitations. The primary limitation of our approach is that we rely on an assumption that the average treatment effect seen in the clinical trials generalizes to the observational data. One way this could be violated is if there is a significant mismatch in the patient population and there is a heterogeneous treatment effect. In that case, it is possible to see different effect directions in the observational data than the randomized trial even if the non-experimental methods are functioning correctly [43, 44]. Another probable mismatch between the observational data and the clinical trials is that there is frequent underreporting of outcomes in our observational datasets because they rely on billing records for adverse events. This is especially the case for non-serious outcomes such as nausea or rashes. Such underreporting would cause the estimated rate of adverse events to be lower in our observational data than in clinical trials A third potential cause is that the clinical trial might not provide a correct effect estimate due to poor internal clinical trial quality (such as improper blinding, poor randomization, and publication bias). For all of these potential causes of different effect estimates, our primary mitigation strategy is to focus on the effect directions of hazard ratios. The benefit of effect directions is that they intrinsically require greater error to change, especially when the effect magnitude is large. Hazard ratios additionally increase resilience by making analysis more resilient to changes in the base rate of the event, whether due to population differences or outcome reporting changes. One piece of evidence that this mitigation strategy is somewhat successful is that we observe much greater concordance between non-experimental methods and clinical trials than what could be achieved by random chance. However, we do expect this mitigation strategy to be imperfect, and differences in the underlying effects should cause us to underestimate the performance of non-experimental methods.

Our work also has several secondary limitations. First, our approach is only able to evaluate methods for detecting average treatment effects because our ground truth is in the form of average treatment effects. We are simply unable to evaluate how effective methods can detect heterogeneous treatment effects. A second additional limitation is that our evaluation strategy simultaneously probes both the statistical method and the observational healthcare data resource used, in that we would only expect high concordance when both are of high quality. This is frequently a disadvantage, in that it can be hard to understand the particular cause of poor concordance. However, in some circumstances, this can be an advantage: *TrialProbe* can help identify potential issues associated with the observational dataset itself (e.g., the underreporting of side effects such as nausea). *TrialProbe* could also be used to probe and contrast different observational datasets, e.g., one could seek to contrast one statistical method applied to a cohort extracted from Optum’s de-identified Clinformatics Data Mart Database compared to the same statistical method applied to a cohort extracted from an alternative observational data resource. Third, our reference set is a biased sample of true drug effects due to selection bias, caused by a combination of publication bias (in the form of trials not reporting results to clinicaltrials.gov) and our requirement for drug prescriptions in our observational data. In particular, it is probably the case that studies that result in significant quantities of adverse events are halted and those drugs are then infrequently (or not at all) used in clinical practice, resulting in our work underestimating the “true” adverse event rates of various drugs. This would in turn mean that the empirical Bayes based subsets that try to identify effects of a particular strength will incorrectly contain stronger effects than expected. However, this should not affect our estimated concordance between non-experimental methods and clinical trials within a particular subset, as we only compare effect directions and not effect magnitudes. Finally, one other disadvantage of our current approach is that the same prior is learned for all log-odds ratios; this presupposes that the selection of effects we consider are relevant to each other. This may not necessarily be the case; for example, chemotherapy drugs will typically have much stronger side effects than other drugs. Not accounting for these differences might cause us to underestimate the effect sizes for high risk drugs like chemotherapy drugs and underestimate the effect sizes for less risky medications. A refinement of the approach would be to stratify effects into groups [45] and learn a separate prior for each group, or to apply methods for empirical Bayes estimation in the presence of covariate information [46].

## Conclusion

We propose an approach for evaluating non-experimental methods using clinical trial derived reference sets, and evaluate three commonly used non-experimental study methods in terms of their ability to identify the known relationships in a commonly used claims dataset. We find that adjustment significantly improves the ability to correctly recover known relationships, with propensity score matching performing particularly well for detecting large effects.

We make *TrialProbe*, i.e., the reference set as well as the procedure to create it, freely available at https://github.com/som-shahlab/TrialProbe. *TrialProbe* is useful for benchmarking observational study methods performance by developers of the methods as well as for practitioners interested in knowing the expected performance of a specific method on the dataset available to them.

## Data Availability

Our code is available at https://github.com/som-shahlab/TrialProbe. The source clinical trial records can be found at clinicaltrials.gov. The data we used in our case study, Optum’s Clinformatics Data Mart Database, is not publicly available as it is a commercially licensed product. In order to get access to Optum’s Clinformatics Data Mart Database, it is generally necessary to reach out to Optum directly to obtain both a license and the data itself. Contact information and other details about how to get access can be found on the product sheet [39]. Optum is the primary long term repository for their datasets and we are not allowed to maintain archive copies past our contract dates.

## Footnotes

1 Such comparisons make sense when there is imperfect compliance to treatment and one is not interested in intention-to-treat effects.

2 Computed with a pseudocount adjustment to deal with zero cell counts, that is, $\textrm{exp}(\widehat{\omega }^{\text {sample}}_i)= \left( {(X_{A,i}+0.5)/(Y_{A,i}+1)}\right) \big /\left( {(X_{B,i}+0.5)/(Y_{B,i}+1)}\right) .$

3 In other words, the investigator does not adjust for any possible confounders.
